# Glutamine induces remodeling of tight junctions in Caco-2 colorectal cancer cell

**DOI:** 10.1007/s12032-022-01896-5

**Published:** 2022-12-02

**Authors:** Ching-Ying Huang, Ji-Kai Chen, Wei-Ting Kuo

**Affiliations:** 1 Department of Food Science and Biotechnology, National Chung Hsing University, 145 Xingda Rd, South Dist, Taichung, Taiwan; 2 Graduate Institute of Oral Biology, College of Medicine, National Taiwan University, Taipei, Taiwan; 3 Department of Dentistry, National Taiwan University Hospital, Taipei, Taiwan

**Keywords:** Glutamine, Tight junction, Zonula occludens (ZO)-1, Colorectal cancer, Transepithelial resistance

## Abstract

Malignant cells often exhibit significant metabolic alterations, including the utilization of different nutrients to meet energetic and biosynthetic demands. Recent studies have shown that glutamine can support primary colorectal tumor growth and also serve as an alternate energy source during distant metastasis under glucose-limited conditions. However, the overall effects of glutamine on cancer cell physiology are not completely understood. In this study, we investigated how glutamine impacts epithelial integrity in colorectal cancer cells under glucose deprivation. Human colorectal cancer (Caco-2) cells were grown to confluency in transwells and cultured in glucose/pyruvate-free DMEM with various glutamine concentrations (0–50 mM). Cell viability was assessed, and monolayer integrity was examined in terms of transepithelial resistance (TER) and paracellular permeability. Tight junction (TJ) component proteins were examined by immunofluorescence staining and Western blotting. A dose-dependent decrease in TER was observed in Caco-2 cells, but paracellular permeability was not affected after 24 h incubation with glutamine. At the same time, the TJ proteins, zonula occludens (ZO)-1 and Claudin-1, showed lateral undulations and punctate staining patterns accompanied by enlargement of cellular and nuclear sizes. Furthermore, decreased protein levels of ZO-1, but not claudin-1, were found in detergent-insoluble cellular fractions. Notably, the decreased TER and alterations in TJ structure were not associated with cell viability changes. Moreover, the addition of glutamate, which is produced by the first step of glutamine catabolism, had no impact on TER. Our results suggested that the enteral glutamine may play an important role in the regulation of TJ dynamics in colorectal cancer cells.

## Introduction

A single nutrient may act as a double-edged sword under physiological and pathophysiological conditions. For instance, the most abundant amino acid in the body, glutamine (GLN), is utilized under normal conditions as a nitrogen donor for nucleotide biosynthesis and a carbon source for oxidative phosphorylation. It also plays important roles the synthesis of proteins and amino sugars [1]. However, GLN can also be utilized by cancer cells to promote oncogenic processes.

Malignant cells often exhibit significant metabolic reprogramming to meet the energetic and biosynthetic demands placed on them by the harsh microenvironment [2]. As such, cancerous colonic cells uniquely employ dual routes of nutrient uptake (from the blood supply and lumen). In addition, the Warburg effect is a well-known pro-survival mechanism observed in many tumor cells wherein aerobic glycolysis is up-regulated [2, 3]. Besides providing energy, the metabolic changes in cancer cells can have other important effects on cell physiology. Along these lines, we previously showed that glycolytic pyruvate confers resistance to RIP-dependent necroptosis in hypoxic colorectal cancer cells by directly scavenging mitochondrial free radicals [4]. Based on this and other mechanisms, several recent studies have suggested that targeting glycolysis may be an attractive therapeutic strategy [2, 5–7]. Cancer cells can also utilize both GLN and glucose (via glutaminolysis and glycolysis, respectively) as nutrients to support cell proliferation and death resistance [2, 8]. In fact, some tumor cells may become addicted to glutaminolysis for the synthesis of biomass and proliferation [9]. GLN enters cancer cells via several glutamine transporter families (SLC1, 6, 7, and 38) [10], after which it is converted to glutamate by mitochondrial glutaminase; the glutamate is subsequently deaminated by glutamate dehydrogenase to form alpha-ketoglutarate. The utilization of this glutamine-derived alpha-ketoglutarate in the TCA cycle is called anaplerotic glutamine metabolism. Recent studies have demonstrated that combined glucose and glutamine depletion results in metabolic reprogramming and growth inhibition, suggesting a potential strategy of targeting metabolic processes in cancer therapies [8, 11, 12]. However, the glutamine-mediated mechanisms involved in modulating cancer cell behaviors remain incompletely understood, especially in the context of glucose depletion.

The human colorectal carcinoma Caco-2 cell line is a well-established model for studies on tight junctions (TJs) and cancer biology. These cells display apical-basal polarity and are connected by intercellular TJs, which consist of transmembrane (occludin, claudins, and junction adhesion molecule) and cytoplasmic zonula occludens (ZO) proteins. In contrast to the transmembrane proteins, which transport solutes and water or seal the junction, the ZO-1 protein was thought to act as a scaffold, anchored to the actin cytoskeleton [13, 14]. Epithelial cancer cells with an invasive phenotype have typically acquired mesenchymal features, such as high motility, through the process known as epithelial–mesenchymal transition (EMT). During EMT, transcriptional reprogramming diminishes the junctions and apical-basal polarity of epithelial cells and causes the cytoskeleton to undergo reorganization [15]. Several lines of evidence support the idea that an early event in EMT is the dissolution of TJs, which is marked by reduced claudin and occludin expression as well as diffusion of ZO-1 away from cell–cell contacts. Intriguingly, glutaminolysis has been linked to EMT in cancer cells [16]. Moreover, GLN was shown to induce phosphorylation of STAT3, which modulates gene transcription and promotes cancer cell metastasis, independent of its metabolic effects [17]. However, the effects of GLN on TJ remodeling and the underlying mechanisms remain largely unknown. In this study, we elucidated the effects of GLN on TJs remodeling in colorectal cancer cells under glucose and pyruvate deprivation.

## Subjects and methods

### Cell lines and cell culture

The human colorectal cancer cell line, Caco-2, was purchased from ATCC/Bioresource Collection and Research Center (BCRC). Caco-2 cells were maintained in standard Dulbecco’s modified Eagle’s medium (DMEM; Invitrogen, Grand Island, NY, USA) containing 5 mM glucose. The media were supplemented with 10% fetal bovine serum, 15 mM HEPES, antibiotics (100 U/mL penicillin, and 0.1 mg/mL streptomycin) (Sigma, St. Louis, MO, USA). The cells were used between passages 21 and 27 and were grown at 37 °C with 5% CO_2_ under a humidified atmosphere. To test the role of GLN in cell behaviors, cells were incubated in glucose-free and pyruvate-free DMEM (Invitrogen) supplemented as above and treated with 0–50 mM glutamine for 24 h. Hypoxic (Hx) challenge was performed using a modular incubator chamber (Billups-Rothenberg, Del Mar, CA, USA) by infusion of 5% CO_2_ and 95% N_2_ at 10 L/min for 5 min; normoxic (Nx) controls were kept at 5% CO_2_ and 95% air [4].

### Measurement of transepithelial electrical resistance (TER) and paracellular permeability

Cells grown to confluency were subjected to glucose and pyruvate depletion and GLN treatment at the indicated concentrations for 24 h. The TER of monolayers was measured using an electrovoltohmeter (EVOM; World Precision Instruments, Sarasota, FL, USA). Paracellular permeability was assessed by apical-to-basal transport of a dextran (MW3000)-conjugated fluorescein (Invitrogen) probe, as described previously [4].

### Immunofluorescence staining of TJ structures

Cells were cultured with or without GLN in glucose/pyruvate-free medium for 24 h. The treated cells were fixed with 4% paraformaldehyde for 1 h on ice, and the reaction was quenched with 50 mM NH_4_Cl in PBS for 10 min at room temperature. After blocking with 0.1% bovine serum albumin (BSA) in PBS for 1 h, monolayers were incubated with polyclonal rabbit anti-human ZO-1 (1:25, Thermo Fisher Scientific, MA, USA) or claudin-1 (1:10, Thermo Fisher Scientific) antibody in permeabilizing buffer (0.05% saponin, and 0.1% BSA in PBS) for 1 h. Cells were then incubated with goat anti-rabbit IgG conjugated to Alexa 488 (1:1000, Thermo Fisher Scientific) secondary antibody for 1 h in the dark, followed by staining with a Hoechst’s dye to visualize cell nuclei. The slides were mounted with aqueous mounting media and viewed under a Zeiss fluorescence microscope.

### Quantification of TJ undulations

Quantification of TJ undulations was performed on cells subjected to ZO-1 immunofluorescence staining. The actual junction length was divided by the distance between tri-cellular junctions to yield the TJ length ratio. Individual images were considered as single measurements. Images were analyzed using ImageJ software [18].

### Western blotting

Cells were scraped from the plate and incubated in ice-cold complete RIPA buffer, then sonicated and centrifuged. The protein concentration of the lysate supernatant was adjusted to 5 mg/ml and diluted at a 1:1 (vol:vol ratio) in 2 × electrophoresis sample buffer containing 2% (w/v) SDS, 100 mM DTT, and 62.5 mM Tris/HCl (pH 6.8). Samples were then heated to 95 °C in a heat block for 5 min and stored at −20 °C until use for immunoblotting.

The extracted proteins were separated by SDS-PAGE, and then the resolved proteins were electrotransferred onto a membrane. After blocking with 5% non-fat milk in TBS, the membrane was incubated with monoclonal mouse anti-ZO-1 (1:500) or anti-Claudin-1 (1:125) antibody (Thermo Fisher Scientific) at 4 °C overnight. A monoclonal mouse anti-β-actin (1:10,000, Sigma-Aldrich, MA, USA) was also used to verify equal loading of samples. Membranes were washed with 0.1% Tween 20 in TBS and incubated with horseradish peroxidase-conjugated goat anti-rabbit or anti-mouse IgG (1:1000, Cell Signaling Technology, MA, USA). Band intensity was quantified by photoimage analysis.

### MTT assay

The tetrazolium dye MTT [3-(4,5-dimethylthiazol-2-yl)-2,5-diphenyltetrazolium bromide] assay (Cayman Chemical, Ann Arbor, MI) was used for cell viability analysis, according to the manufacturer’s instructions. The principle of the MTT assay is that it measures the metabolic activity of NAD(P)H-dependent cellular oxidoreductase enzymes by a colorimetric reaction. The absorbance of the product (~ 570 nm) reflects the number of viable cells present.

### Statistical analysis

All values are expressed as mean ± SEM. Data were compared by one-way ANOVA followed by a Student-Newman-Keul test or ANOVA followed by Fisher’s least significant difference test using Prism (GraphPad) or Sigma Plot software. Statistical significance was set at *p* < 0.05.

## Results

### Apical administration of GLN reduces transepithelial resistance (TER) but not paracellular permeability in human colorectal cancer Caco-2 cells

**Fig. 1:**
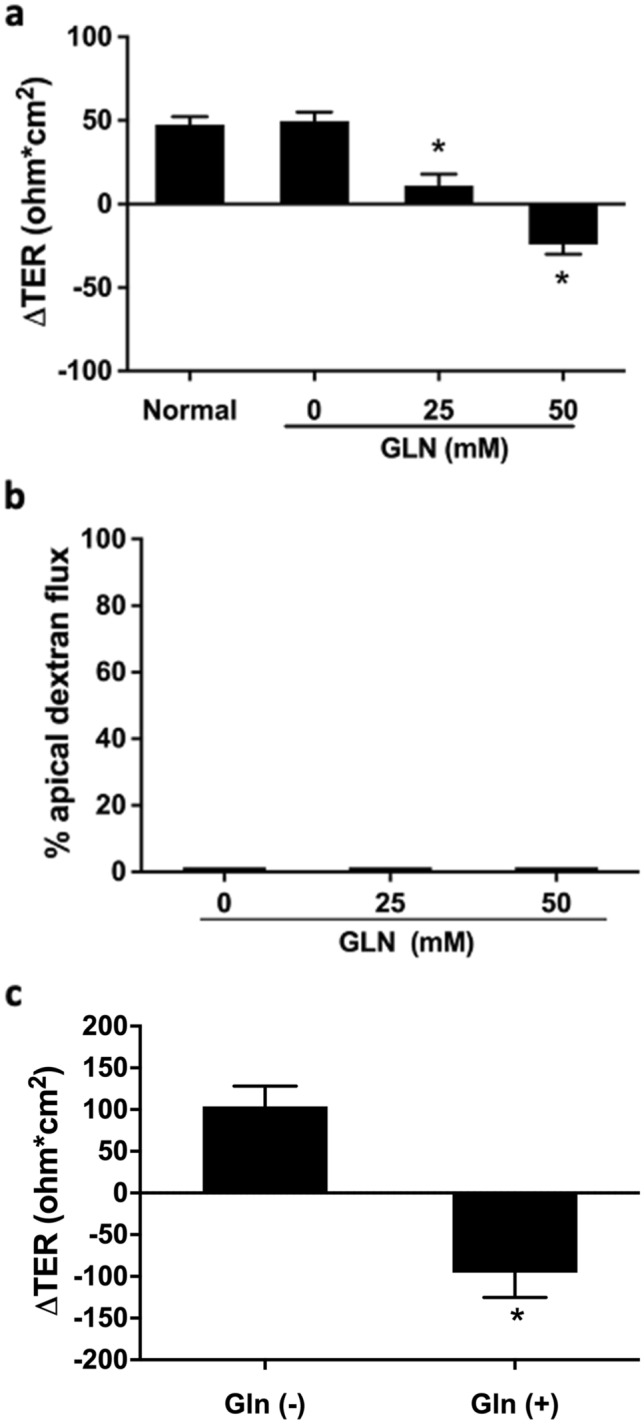
**Apical addition of GLN caused a reduction of transepithelial resistance (TER) but not paracellular permeability in human colorectal cancer cells.** Epithelial integrity was measured in Caco-2 cells incubated in glucose/pyruvate-free medium with apical **(a**, **b)** or basolateral **c** treatment of GLN at different concentrations for 24 h. **a** Apical addition of glutamine caused a significant decrease in TER values, but it did not affect **b** paracellular apical-to-basolateral dextran flux. **c** TER was also decreased in Caco-2 cells with basolateral GLN treatment. *p < 0.05 versus 0 mM GLN or GLN (-) groups (*n* = 6/group)

Human colorectal carcinoma Caco-2 cells were seeded in transwells, and TER was monitored every 2–3 days. When the TER measurements reached a steady plateau, TJs were considered to be fully differentiated. To identify the impact of GLN on epithelial characteristics, Caco-2 cells with fully differentiated TJs were incubated in glucose/pyruvate-free medium, and 0–50 mM GLN was applied to the apical side for 24 h. Deprivation of glucose and pyruvate caused no significant changes in TER values, as compared to cells cultured in normal complete medium (Fig. 1a). Apical treatment of GLN to Caco-2 cells dose-dependently reduced TER measurements (Fig. 1a). However, no significant difference of apical-to-basolateral dextran flux was seen between cells without or with GLN treatment (1.392 ± 0.017% and 1.379 ± 0.016%, respectively; Fig. 1b). We then tested whether basolateral treatment of GLN also altered TER. Glucose/pyruvate-free medium without or with GLN was added to the basolateral compartment of the transwells, and TER was measured (Fig. 1c). Treating the Caco-2 cells with GLN on the basolateral side also decreased TER values, suggesting that dual routes of GLN uptake may contribute to the reduction of TER.

### GLN alters cell morphology and remodels tight junction complexes in Caco-2 cells

**Fig. 2:**
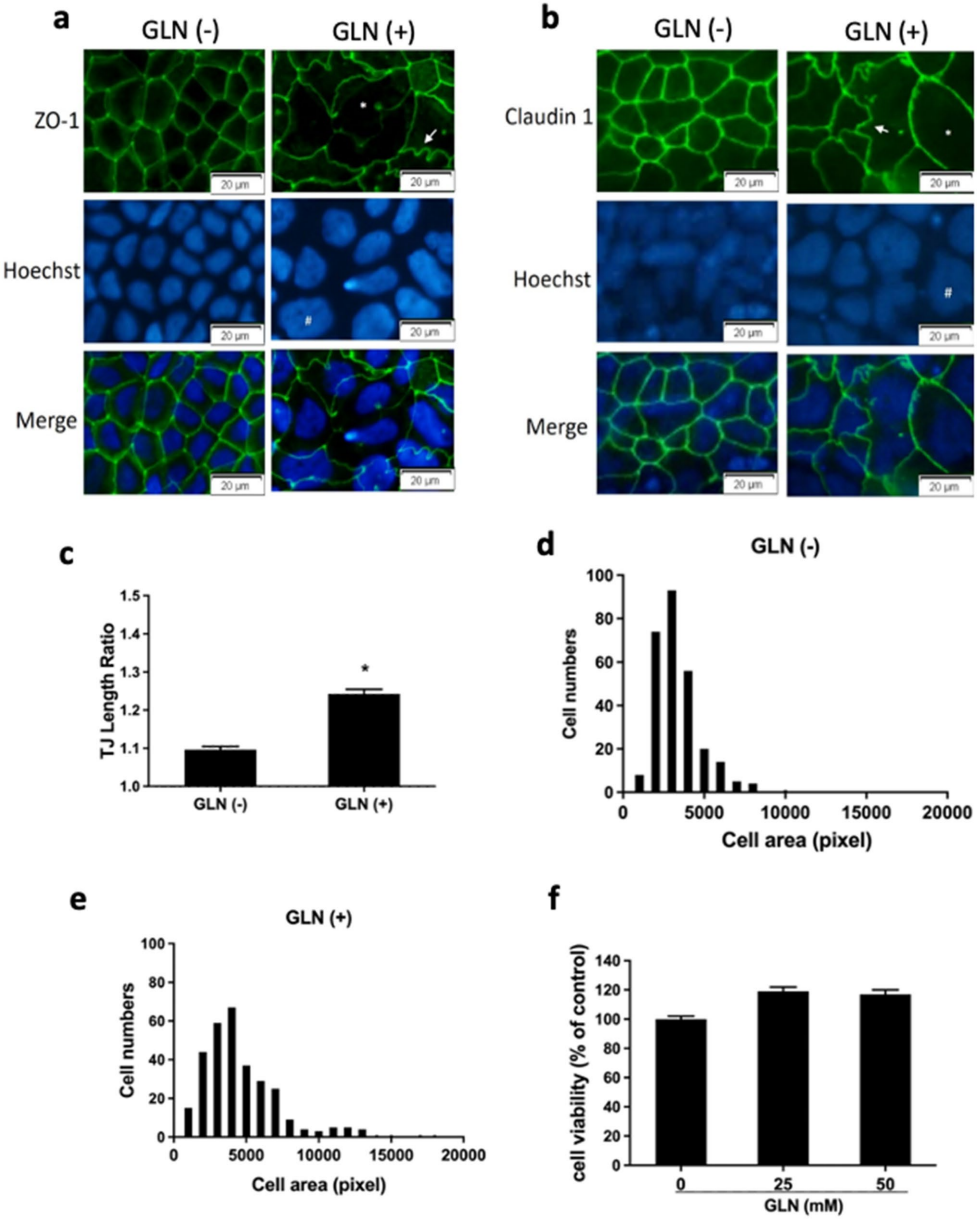
**Caco-2 cells exhibit characteristic TJ abnormalities after GLN treatment.** Immunofluorescence staining of **a** ZO-1 or **b** Claudin-1 (green) was performed, and cell nuclei (blue) were stained with Hoechst’s dye following incubation of Caco-2 cells without or with apical GLN for 24 h. A typical chicken-wire pattern of TJs was observed in GLN-free (GLN(-)) group. Undulating and punctate ZO-1 and claudin-1 staining (arrows) were observed in cells exposed to 50 mM GLN (GLN( +)). Enlargements of cellular and nuclear size were found in Caco-2 cells treated with GLN (#). **c** The lateral undulation of epithelial cells was quantified by dividing the actual junction (ZO-1) length by a straight distance between tri-cellular junctions (to yield the TJ length ratio). The TJ length ratio was significantly higher in the GLN ( +) group. Distributions of epithelial cell sizes in **d** GLN(-) and **e** GLN( +) groups were quantified by measuring relative cell area of immunofluorescence images, as represented in **(a)**. **f** Caco-2 cell viability was determined by MTT assay. No significant difference in cell viability was found between GLN (-) and GLN ( +) groups. *p < 0.05 vs. GLN (-) (**a**–**c**, *n* = 6/group; **d** & **e**, *n* = 150 cells/group; **f**, *n* = 8/group)

**Fig. 3:**
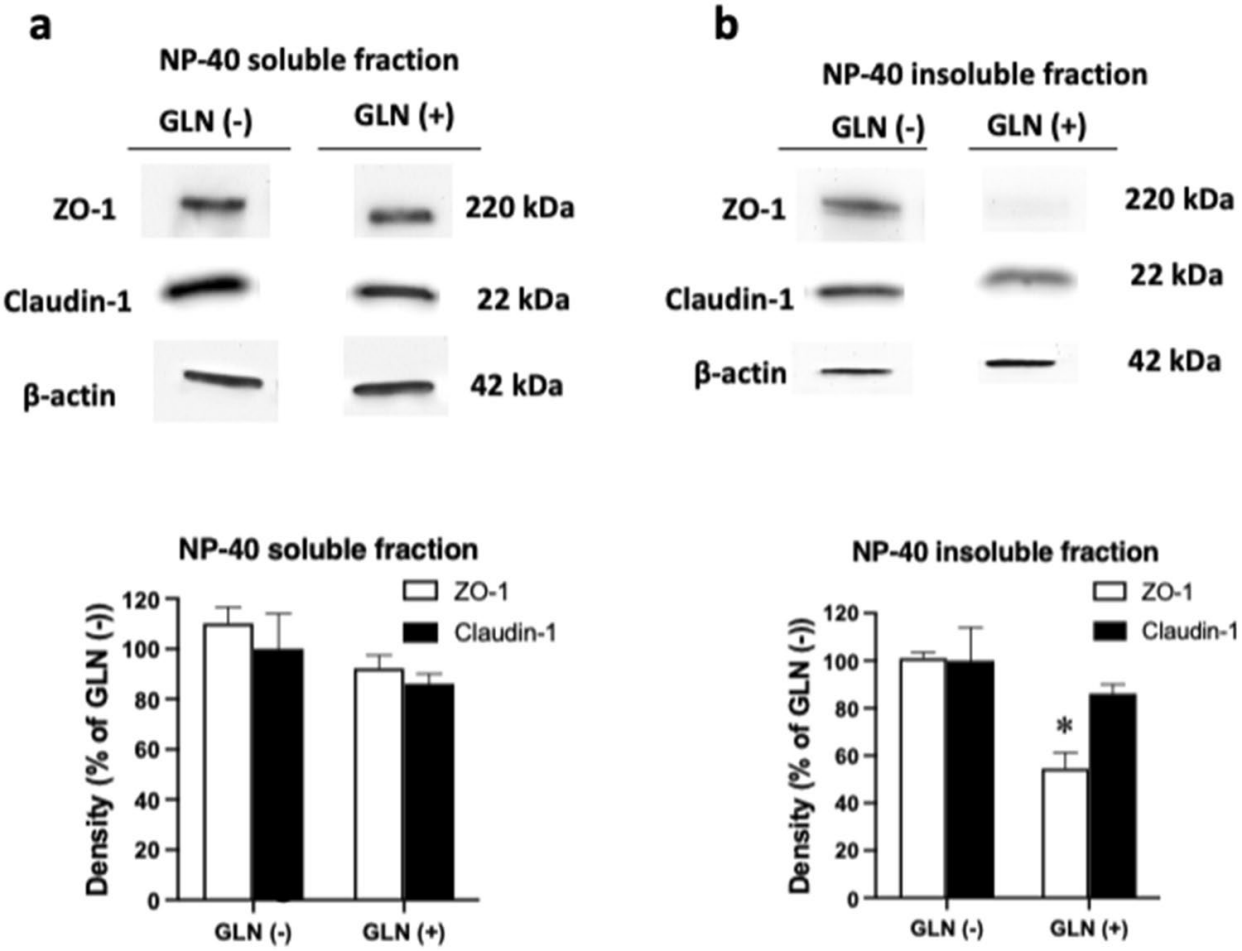
**Alterations in TJ complexes of Caco-2 cells following GLN administration**. Human Caco-2 cells were treated without or with apical GLN (50 mM) for 24 h. Different cellular fractions were collected for Western blotting, including the **a** detergent-soluble fraction and **b** detergent-insoluble fraction. Western blots of ZO-1 and claudin-1 are shown; band intensity was quantified by densitometric analysis. Decreased levels of ZO-1, but not claudin-1, were found in the detergent-insoluble fraction. *p < 0.05 vs. GLN (-) (*n* = 3–6/ group)

After observing TER defects in GLN-treated Caco-2 cells, we next visualized the TJ complexes by staining for ZO-1 and Claudin-1 in confluent Caco-2 monolayers. ZO-1 plays a pivotal role in anchoring the TJ to the actin component of the cytoskeleton. [19] Of note, a previous study showed that overexpression of Claudin-1 alleviates morphological abnormalities in hypoxia-inducible factor β-deficient Caco-2 cells. [18] Our examination revealed typical chicken-wire patterns of ZO-1 and Claudin-1 staining in Caco-2 cells incubated without GLN under glucose/pyruvate deprivation; linear contacts were observed between cells, and ZO-1 and Claudin-1 were distributed uniformly along cell–cell junctions (Fig. 2a & b). However, immunofluorescence analyses of cells treated with GLN for 24 h showed dramatic morphological abnormalities, characterized by pronounced undulations of ZO-1 and Claudin-1 along an uneven lateral membrane. To further analyze TJ morphology, the TJ length ratio was calculated from images of ZO-1 staining by dividing the actual junction length by the distance between tri-cellular junctions. This analysis revealed an increase of TJ length ratio upon GLN treatment. Distribution of Caco-2 cell size was also quantified by measuring the relative cell areas. The results showed a reduced uniformity of cell size profiles in cultures treated with GLN (Fig. 2d & e). However, the decrease in TER and disorganization of TJs caused by apical administration of GLN were not associated with increased cell death, as evidenced by the MTT assay (Fig. 2f). We further examined the regulation of TJ proteins during GLN-mediated epithelial restructuring. Detergent-soluble fractions of ZO-1 and Claudin-1 were not altered after 24-h apical treatment of GLN (Fig. 3a). However, decreased levels of ZO-1 were found in the detergent-insoluble cellular fractions. The altered morphology (Fig. 2a) and decreased level of ZO-1 (Fig. 3b) may be associated the decreased TER values (Fig. 1a) after GLN treatment.

### GLN-mediated TJ remodeling is uncoupled from GLN conversion to glutamate (GLU)

**Fig. 4:**
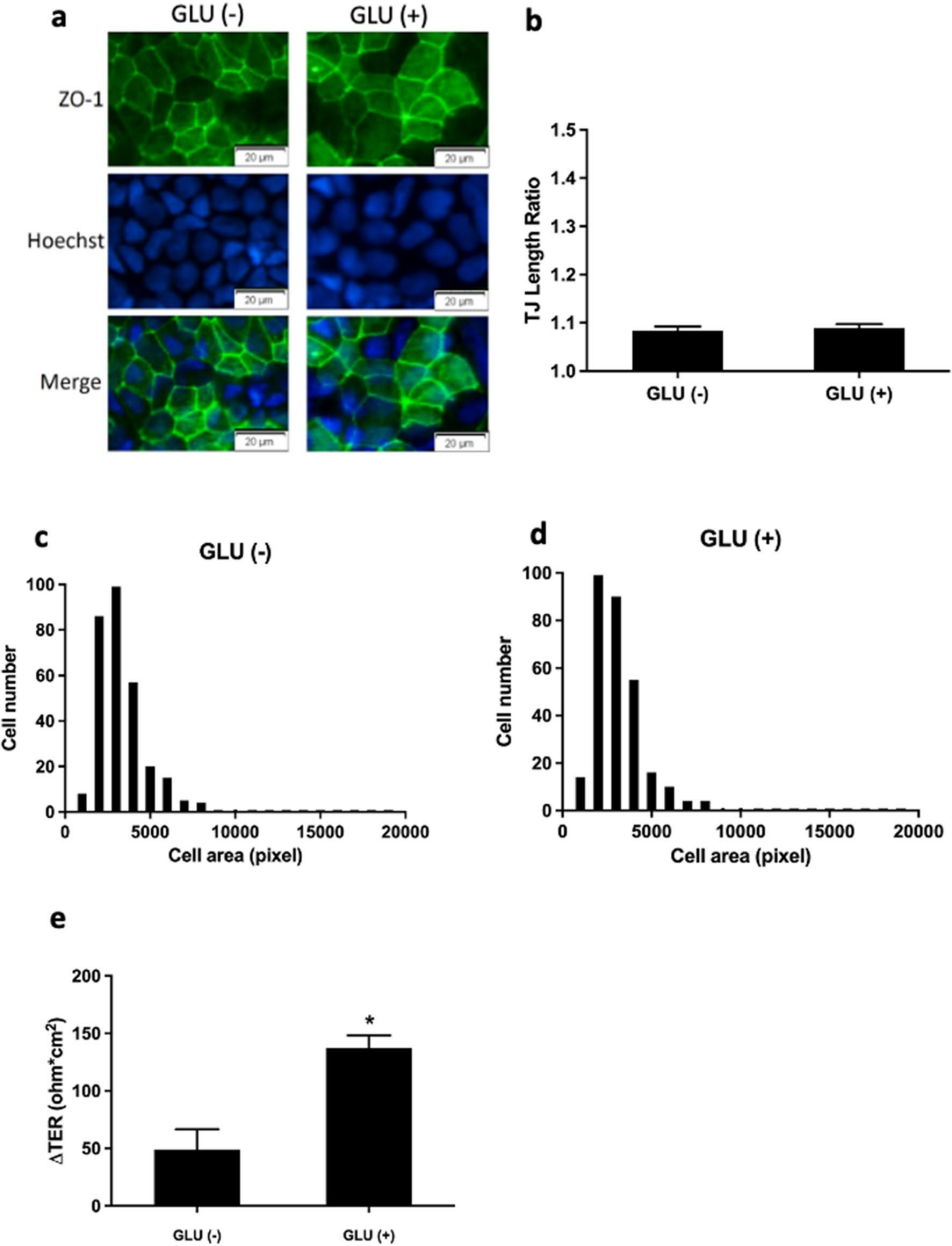
**Apical addition of glutamate (GLU) has no impact on TJ structure in Caco-2 cells.** **a** No changes in ZO-1 (green) structure were observed upon immunofluorescence staining of Caco-2 cells following incubation without or with apical GLU for 24 h. Quantification of **b** TJ length ratio and **c, d** distribution of epithelial cell size in GLU (-) and 50 mM GLU ( +) groups were quantified from immunofluorescence images similar to those shown in (**a**). **e** Transepithelial resistance (TER) was increased in Caco-2 cells after 24-h incubation with apical GLU. *p < 0.05 vs. GLU (-) (**a**, **b**, **e**, *n* = 6 /group; **c**, **d**, *n* = 150 cells /group)

Next, we examined whether the TJ remodeling caused by GLN was related to its metabolism. To do so, we performed the same experiments, replacing GLN with GLU. Addition of GLU had no effect on TJ or cellular morphologic features, as evidenced by ZO-1 staining, TJ length ratio, and cell size distribution (Fig. 4a–d). Additionally, TER was unchanged in Caco-2 cells upon addition of GLU under in glucose/pyruvate deprivation (Fig. 4e). These data suggested that GLN-mediated TJ remodeling might be regulated by signaling events and not metabolic effects.

### GLN cannot rescue Caco-2 cells from hypoxia-induced epithelial integrity loss

**Fig. 5:**
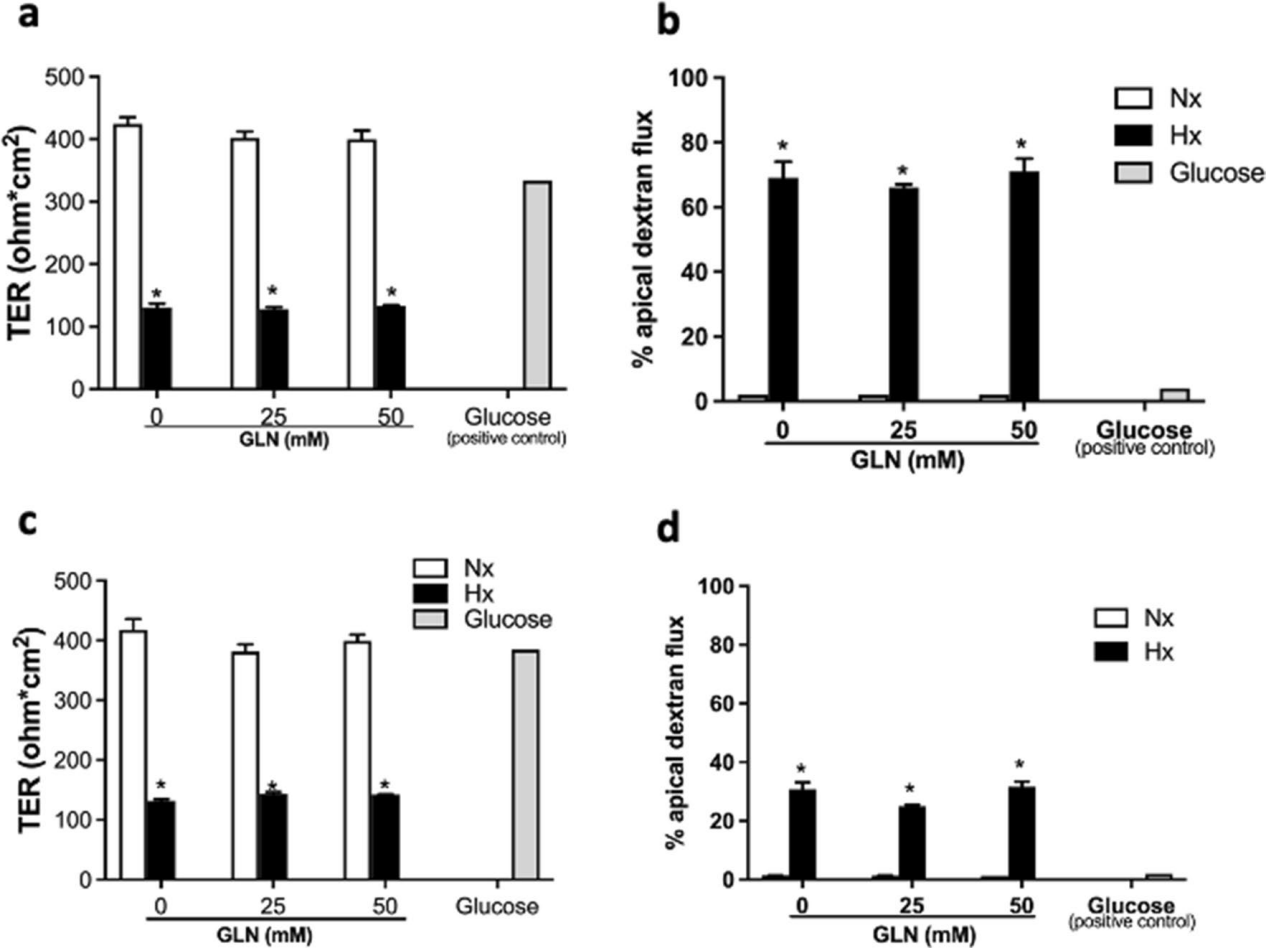
**GLN does not restore hypoxia-induced loss of epithelial integrity in human colorectal cancer cells.** Caco-2 cells were exposed to normoxia (Nx) or hypoxia (Hx) for 16 h. Neither (**a**, **b**) apical nor (**c**, **d**) basolateral addition of GLN restored the Hx-induced (**a** & **c**) TER drop or (**b** & **d**) paracellular dextran flux in Caco-2 cells. *p < 0.05 vs. GLN (-) (*n* = 8 /group)

We next evaluated whether GLN could maintain TJ integrity under a hypoxic challenge. Caco-2 cells were exposed to hypoxia for 16 h, along with apical administration of various concentrations of GLN. A significant drop of TER and coincident increase of paracellular dextran flux was found in the hypoxia-exposed cultures, regardless of the presence of GLN (Fig. 5).

## Discussion

In this study, we found that apical administration of high-dose GLN (25 or 50 mM) reduced TER in human colorectal cancer Caco-2 cells but did not alter paracellular permeability. The epithelial cells exhibited lateral undulations and punctate staining of TJ structural proteins accompanied by enlargement of cellular and nuclear size after GLN treatment. Furthermore, levels of ZO-1 were decreased but Claudin-1 levels were unchanged in detergent-insoluble cellular fractions, suggesting a possible role for ZO-1 in GLN-mediated TJ remodeling. The decrease of TER and altered TJ structure was not associated with changes in cell viability.

The eventual distribution of GLN in the gastrointestinal tract after ingestion is not well understood. Early studies on normal human volunteers fed a test meal containing 50 g purified bovine albumin showed that luminal GLN concentrations may be as high as 5 mmol/L [20, 21]. Daily supplementation of GLN in oral/enteral or parenteral nutrition sources may vary from a fixed dose of 20–35 g/24 h to an adjusted dose of < 1.0 g (dipeptide forms) per kg of body weight [1]. Moreover, metabolomics and LC–MS analyses revealed that GLN concentrations were lower in the tumor core compared with the peripheral regions of melanoma xenografted tumors [22] and human pancreatic tumor specimens [23]. Furthermore, the dual routes of GLN uptake by colorectal cancer cells add further complexity to show the distribution of GLN in the tumor microenvironment affects cancer progression.

In line with our findings, recent studies demonstrated that the depletion of both glucose and GLN resulted in metabolic reprogramming and growth inhibition, suggesting an alternative therapeutic strategy of inhibiting metabolism in malignant cells [8, 11, 12]. The depletion of glucose in the tumor microenvironment may drive nutrient competition between tumor cells and T cells that promotes cancer progression [24]. Moreover, an investigation detailing how nutrients partition in the tumor microenvironment showed that cell-intrinsic programs drive the respective preferential acquisition of glucose and GLN by immune and cancer cells [25]. These findings are consistent with the results of our current study, which showed that GLN cannot support Caco-2 cell survival under hypoxic challenge (Fig. 5); instead, colorectal cancer cells may require glucose as an energy source to support survival and proliferation under hypoxia [4, 26, 27]. Together, these previous studies and our current findings suggest that the supply of nutrients in tumor microenvironment may contribute to tumor heterogeneity and dynamic behaviors.

TJ protein complexes greatly influence the permeability of epithelia, allowing the plasma membranes of adjacent cells to form contacts that stringently occlude extracellular factors. Early studies analyzing biopsies from colorectal cancer demonstrated that the level of ZO-1 is frequently reduced in primary colorectal cancer as compared to the normal epithelium in patients with liver metastasis (20.8%); furthermore, ZO-1 is often re-expressed in liver metastasized cancers (79.2%) [28]. In an in vivo animal model of lung cancer, the reduction of ZO-1 is an early event, which occurs prior to EMT [29]. Moreover, the down-regulation of ZO-1 leads to increased motility in pancreatic cancer cells. [30] Phosphorylation of perijunctional myosin light chain (MLC) by myosin light chain kinase (MLCK) [31–33] or Rho kinase (ROCK) is sufficient to increase epithelial permeability via activating actomyosin contraction and TJ remodeling. Clinically, MLCK and ROCK inhibitors have been used as migrastatic drugs for cancer therapy, but their use is limited by drug toxicity [34]. Although we did not probe MLC phosphorylation in our experiments, our results suggest that further studies on the role of MLC in GLN-mediated TJ remodeling may be warranted.

In conclusion, we found that under conditions of glucose and pyruvate deprivation, treatment of Caco-2 colorectal cancer cells with GLN decreased the level of ZO-1 and caused loss of epithelial integrity, independent of cell death and GLN conversion to GLU. These results suggest a pleiotropic role for GLN that includes the breakdown of cell–cell contacts and may be relevant to EMT in colorectal cancer.

## Data Availability

The datasets generated during and/or analyzed during the current study are available from the corresponding author on reasonable request.
